# Phosphorylation of vaccinia-related kinase 1 at threonine 386 transduces glucose stress signal in human liver cells

**DOI:** 10.1042/BSR20200498

**Published:** 2020-04-23

**Authors:** Kosuke Yokobori, Yuu Miyauchi, Jason G. Williams, Masahiko Negishi

**Affiliations:** 1 Pharmacogenetics section, Reproductive and Developmental Biology Laboratory, National Institute of Environmental Health Sciences, National Institutes of Health, Research Triangle Park, North Carolina 27709, U.S.A.; 2 Mass Spectrometry Research and Support Group, Epigenetics and Stem Cell Biology Laboratory, National Institute of Environmental Health Sciences, National Institutes of Health, Research Triangle Park, North Carolina 27709, U.S.A.

**Keywords:** c-Jun, Glucose stress signaling, phosphorylation/dephosphorylation, Plakophilin-2, Vaccinia-related kinase1

## Abstract

Vaccinia-related kinase 1 (VRK1) is a chromatin-associated Ser-Thr kinase that regulates numerous downstream factors including DNA repair as well as stress factors c-Jun and p53. Both c-Jun and p53 are phosphorylated at Ser63 and Thr18, respectively, in response to low glucose (40 mg/dl of medium) but not high glucose (140 mg/dl of medium) in human hepatoma-derived Huh-7 cells. Here, we have determined the molecular mechanism by which VRK1 phosphorylates these residues in response to glucose in Huh-7 cells. Human VRK1 auto-phosphorylates Ser376 and Thr386 in *in vitro* kinase assays. In Huh-7 cells, this auto-phosphorylation activity is regulated by glucose signaling; Thr386 is auto-phosphorylated only in low glucose medium, while Ser376 is not phosphorylated in either medium. A correlation of this low glucose response phosphorylation of Thr386 with the phosphorylation of c-Jun and p53 suggests that VRK1 phosphorylated at Thr386 catalyzes this phosphorylation. In fact, VRK1 knockdown by siRNA decreases and over-expression of VRK1 T386D increases phosphorylated c-Jun and p53 in Huh-7 cells. Phosphorylation by VRK1 of c-Jun but not p53 is regulated by cadherin Plakophilin-2 (PKP2). The PKP2 is purified from whole extracts of Huh-7 cells cultured in low glucose medium and is characterized to bind a C-terminal peptide of the VRK1 molecules to regulate its substrate specificity toward c-Jun. siRNA knockdowns show that PKP2 transduces low glucose signaling to VRK1 only to phosphorylate c-Jun, establishing the low glucose-PKP2-VRK1-c-Jun pathway as a glucose stress signaling pathway.

## Introduction

Vaccinia-related kinase 1 (VRK1) is a member of the VRK subfamily within the casein kinase superfamily [1]. VRK1, a chromatin kinase, is well known to regulate various cell maintenance and survival functions in response to DNA damages caused by insults such as UV exposure [2]. VRK1 plays roles in spermatogenesis in mice and its mutations are linked to developments of neurodegenerative diseases such as amyotrophic lateral sclerosis (ALS) [3–7]. In addition, VRK1 has been suggested to regulate stress response factors such as p53 and c-Jun and transcription factors including cAMP response element binding protein (CREB) and farnesoid X receptor (FXR) [8–11]. However, the cell signals to which VRK1 responds to activate these factors remains unknown. Recently, it was reported that VRK1 responds to glucose leads to regulate gluconeogenesis through phosphorylation of pregnane X receptor (PXR) in human hepatoma-derived HepG2 cells [12]. Thus, VRK1 may be a glucose response signal kinase that regulates hepatic energy metabolism. Since stress factors p53 and c-Jun have also been implicated in hepatic gluconeogenesis and diabetes [13–15], we examined whether glucose regulates these factors and, if so, whether VRK1 transduces this glucose signal. Moreover, the molecular mechanism of the VRK1-mediated signal transduction was investigated. First, the experimental basis for this investigation was established by finding that both p53 and c-Jun were phosphorylated in Huh-7 cells when cultured in low but not high glucose media and that VRK1 catalyzed this low glucose response phosphorylation.

VRK1 possesses high auto-phosphorylation activity in *in vitro* kinase assays [16]. However. VRK1 should repress phosphorylation of these residues to acquire its signal response capability in cells *in vivo.* Therefore, identifying auto-phosphorylated residues of VRK1 was a prerequisite to characterizing VRK1 as a glucose signal transducer in cells. For this, VRK1 auto-phosphorylated in *in vitro* assays was subjected to mass spectrometry. Phospho-peptide antibodies were produced to examine VRK1 phosphorylated at these residues in cells. VRK1 contains a C-terminal random coil that is looped out from the catalytic domain [16]. A C-terminal region of this loop is known to regulate the auto-phosphorylation activity of VRK1 [16]. Expression vectors bearing various mutations within this region were constructed to further examine the molecular basis that regulates VRK1 activity. Utilizing a C-terminal peptide as an affinity bait, proteins that bind in response to low glucose were purified from whole cell extracts of Huh-7 cells. The resultant proteins were investigated as candidates for a glucose response factor that regulates VRK1 activity to phosphorylate c-Jun and p53. This manuscript presents evidence in support of the molecular mechanism by which VRK1 mediates glucose signaling to downstream stress factors.

## Materials and methods

### Antibodies

Rabbit polyclonal antibodies against synthetic phospho-peptide (TEW(pSER)NTQTEEAIQTC) or (TEEAIQ(pTHR)RSRTRKRC) corresponding to residues surrounding Ser376 or Thr386, respectively, for human VRK1 were produced and evaluated by GenScript (Piscataway, NJ, U.S.A.). Antibodies to phosphorylated C-Jun at S63 (9261S), total C-Jun (2315S), total p53 (9282), and total VRK1 (3307S) were obtained from cell signaling technology (Danvers, MA, U.S.A.). Antibody to phosphorylated p53 at T18 (PA5-12660) was obtained from Thermo Fisher Scientific (Waltham, MA, U.S.A.). Antibodies to beta-actin (sc-130656), PKP2 (sc-136039), His (sc-804), GST (sc-459, HRP conjugated), rabbit IgG (sc-2004, HRP conjugated) or mouse IgG (sc-2314, HRP conjugated) were obtained from Santa Cruz Biotech (Dallas, TX, U.S.A.). Antibody to FLAG (A8592-2MG) was obtained from Sigma-Aldrich (St louis, MO, U.S.A.).

### Cell culture and treatment

Human hepatoma-derived Huh-7 cells were cultured in D-MEM (glucose concentration: 450 mg/dl) supplemented with 10% (v/v) heat-inactive fetal bovine serum, 100 units/ml penicillin, and 100 μg/ml streptomycin (hereafter, called DMEM-450) and were maintained at 37°C in a humidified atmosphere with 5% CO_2._ Glucose concentration in the D-MEM was adjusted to 40, 100 and 140 mg/dl (hereafter, called DMEM-40, DMEM-100 and DMEM-140) by mixing D-MEM (no glucose) and DMEM-450. After cultured in DMEN-450 for 24 h, cells were cultured in DMEM-100 without FBS for 24 h. After medium were changed to DMEM-40, DMEM-100 or DMEM-140, cells were cultured for an additional 3 h. DNA damage induction by UV light was performed by UV Stratalinker 1800 (Stratagene, San Diego, CA, U.S.A.). Cells were exposed UV light for 10 min.

### Plasmids

FLAG-VRK1/pcDNA3.1, GFP-VRK1/pEGFP-c1 and GST-VRK1/pGEX4T3 were described previously [12]. PKP2 was amplified using PrimeSTAR Max (TAKARA Bio Inc., Shiga, Japan) from human liver cDNA libraries and cloned using TOPO-TA cloning kit (Thermo Fisher Scientific). Subcloned PKP2 was inserted into a FLAG fusion protein expression vector or GST fusion protein expression vector. Subcloned VRK1 or PKP2 was inserted into a His_6_-SUMO fusion protein expression vector [17]. Proper primer sets were used to introduce deletions or base mutations using PrimeSTAR Max. All constructs were verified by nucleotide sequencing.

### Transfection

For siRNA knockdowns, cells were transfected with ON-TARGETplus SMART pool human VRK1 siRNA, human PKP2 or Non-Targeting pool (Dharmacon Research, Lafayette, CO, U.S.A.) using Lipofectamine RNAiMAX (Thermo Fisher Scientific). After transfection in DMEM-450 for 24 h, cells were cultured in 100 mg/dl glucose and transfected with FLAG-VRK1 by Fugene6 (Promega, Fitchburg, WI, U.S.A.) for 24 h. Cells were cultured in DMEM-40, DMEM-100 or DMEM-140 for 3 h. For co-immunoprecipitation assays, FLAG-PKP2 was transfected with GFP-VRK1 or GFP for cells cultivated in DMEM-100 by Fugene6 after cell seeding for 24 h. Cells were exposed to different glucose concentration for 3 h.

### Immunoprecipitation and Western blot

Whole cell extracts were prepared from cells using Urea lysis buffer (8M Urea, 50 mM Tris-HCl, pH 7.5, 0.1% SDS), IP buffer for anti-phospho VRK1 antibody (20 mM Tris-HCl, pH 7.5, 100 mM NaCl, 1% Triton X (100), 10% Glycerol) or Co-IP buffer (20 mM Tris-HCl, pH 7.5, 500 mM NaCl, 0.5 mM EDTA, 10% glycerol). For immunoprecipitation of phospho-VRK1, whole cell lysates were prepared using IP buffer and then incubated with Dynabeads Protein G conjugated with proper antibody. For co-immunoprecipitation, whole cell lysates were prepared using Co-IP buffer and then incubated with anti-GFP agarose beads. Immunoprecipitated beads were washed three times with lysis buffer. Washed beads were heat-treated in 2× SDS sample buffer (250 mM Tris-HCl, pH 6.8, 2% SDS, 25% glycerol, 0.01% bromophenol blue). Whole cell lysates were prepared using Urea lysis buffer and then heat-treated in 4× SDS sample buffer (500 mM Tris-HCl, pH 6.8, 4% SDS, 50% glycerol, 0.02% bromophenol blue). Proteins were separated in a 10% SDS-polyacrylamide gel and were transferred onto a PVDF membrane. Membranes were blocked with 5% skim milk in TBS containing 0.1% Tween 20 and were incubated with appropriate primary and secondary antibodies. Protein bands were visualized by Western Bright ECL reagents.

### Recombinant protein expression and purification

HisSUMO-VRK1, HisSUMO-PKP2 or GST-VRK1 plasmid were transformed into *Escherichia Coli* BL21CodonPlus (DE3)-RIL (Agilent, Santa Clara, CA) cells. Cells were grown in 10 ml LB medium by shacking at 37°C for 16 h. Five milliliters of culture medium was transfer to 1 L terrific broth medium shaking at 37°C until an OD600 of 0.5–0.7 was attained. The recombinant protein expression was induced by addition of 0.1 mM isopropyl-1-thio-D-galactopyranoside (IPTG) and cells were allowed to further incubate at 10°C for 16 h while shacking. Cells were centrifuged at 1000 × ***g*** for 15 min at 4°C. Cell pellets were re-suspended in lysis buffer (25 mM Tris-HCl (pH 7.5), 100 mM NaCl, and 1 mM DTT) and sonicated for 30 s five times. The lysates were ultra-centrifuge at 167,000 × ***g*** for 45 min. The supernatants were collected to use for further experiments. The supernatants from His-tagged protein expressed samples were incubated with Ni-NTA-agarose beads (Qiagen) and then beads were washed with lysis buffer. Protein was eluted from Ni-beads with lysis buffer containing 400 mM imidazole. Recombinant VRK1 was purified from the HisSUMO-VRK1 fusion by proteolytic cleavage with ULP-1 protease during overnight dialysis with 1000-fold lysis buffer at 4°C. The His-SUMO and ULP-1 protease were removed by passing through a Ni-NTA-agarose column.

### *In vitro* kinase assay

In *in vitro* kinase assays, purified recombinant VRK1 was incubated in 20 mM Tris-HCl, pH 7.5 buffer containing 5 mM MgCl_2_, 150 mM KCl and 5 mCi of γ32P-ATP and/or 30 μM ATP at 37°C for 30 min. Protein was separated on a 10% SDS-polyacrylamide gel and autoradiographed or analyzed by mass-spectrometry or Western blotting.

### Affinity purification

VRK1 bound proteins were immunoprecipitated by anti-GFP agarose beads incubated with whole cell extracts of which GFP-VRK1 or GFP transfected cells were treated with low or high glucose for 3 h. C-terminus of VRK1 bound proteins were immunoprecipitated by C-terminus peptide WT (NTQTEEAIQTRSRTRKRVQK) or 6E (NTQTEEAIQTESETEEEVQE) conjugated Affi-gel10 or Affi-gel15 (Bio-Rad laboratories, Hercules, CA, U.S.A.), respectively, incubated with whole cell extracts of which Huh-7 cells were treated with low or high glucose for 3 h. The beads were washed by Co-IP buffer.

### Mass spectrometry

Proteins were identified by mass spectrometry, similarly to as previously described [18]. Briefly, gel slices were excised and minced manually. Digests were then performed with a ProGest robotic digester (Genomic Solutions, Ann Arbor, MI, U.S.A.) where the gel pieces were destained by incubation in 25 mM ammonium bicarbonate/50% v/v acetonitrile (2 × 15 min). The gel pieces were dehydrated in acetonitrile, followed by drying under a nitrogen stream, and then incubated with 250 ng trypsin (Promega) for 8 h at 37°C. The digests were collected and peptides were re-extracted three times. The extractions were pooled for each sample, lyophilized, and resuspended in 20 µl 0.1% formic acid. Protein digests were analyzed by LC/MS on a Q Exactive Plus mass spectrometer (ThermoFisher Scientific) interfaced with a nanoAcquity UPLC system (Waters Corporation, Milford, MA, U.S.A.) equipped with a 75 µm × 150 mm BEH dC18 column (1.8 µm particle size, Waters Corporation) and a C18 trapping column (180 µm × 20 mm) with 5 µm particle size at a flow rate of 400 nl/min. The trapping column was positioned in-line of the analytical column and upstream of a micro-tee union that was used both as a vent for trapping and as a liquid junction. Trapping was performed using the initial solvent composition. About 5 µl of digested sample was injected onto the column. Peptides were eluted by using a linear gradient from 99% solvent A (0.1% formic acid in water (v/v)) and 1% solvent B (0.1% formic acid in acetonitrile (v/v)) to 40% solvent B over 60 min. For the mass spectrometry, a data acquisition method was employed with an exclusion time of 15 s and an exclusion of +1 charge states. The mass spectrometer was equipped with a NanoFlex source and was used in the positive ion mode. Instrument parameters were as follows: sheath gas, 0; auxiliary gas, 0; sweep gas, 0; spray voltage, 2.7 kV; capillary temperature, 275°C; S-lens, 60; scan range (*m/z*) of 200–2000; 2 *m/z* isolation window; resolution: 70,000; automated gain control (AGC), 2 × 10e5 ions; and a maximum IT of 200 ms. Mass calibration was performed before data acquisition using the Pierce LTQ Velos Positive Ion Calibration mixture (ThermoFisher Scientific). Peak lists were generated from the LC/MS data using Mascot Distiller (Matrix Science, Boston, MA, U.S.A.) and the resulting peak lists were searched using the Spectrum Mill software package (Agilent) against a species limited (human rodent) Swissprot and custom VRK1 protein database. Searches were performed using trypsin specificity and allowed for two missed cleavages. Mass tolerances were 20 ppm for MS scans and 50 ppm for MSMS scans. Serine, threonine, and tyrosine phosphorylation as well as methionine oxidation were allowed as variable modifications. For intact mass analyses, approximately 1 g of purified recombinant VRK1 was injected using the same gradient and instrument parameters described above. Data were processed for intact mass analyses using BioPharma Finder 3.0 (Thermo Fisher Scientific) using the manual processing option of the ReSpectTM (Isotopically Unresolved) function of the software. Software parameters included a Model Mass Range of 10,000–60,0000, a Charge State Range of 10–100, and a Target Mass of 40,000 Da. To analyze candidate binding partners, we calculated normalized spectral counts for proteins associated with VRK1 by dividing the spectral counts from the VRK1 pull-down lanes by the spectral counts from the GFP only pull-down lanes, to prevent division by zero, the value 0.9 (a conservative value to prevent overestimating selective enrichment by VRK1 pull-down) was substituted if the value in the GFP-only pulldown was zero.

### Pull-down assay

GST or GST-VRK1 proteins were purified with glutathione-Sepharose 4B (GE Healthcare, Uppsala, Sweden). GST or GST-VRK1 bound to resin were incubated with HisSUMO-PKP2. Bound proteins were subjected to Western blot assays.

## Results

### Glucose

D-MEM media supplemented with various glucose concentrations from zero, 40, 100, 140 to 450 mg/dl, hereafter called DMEM-zero, DMEM-40, DMEM-100, DMEM-140 and DMEM-450, respectively. Based on blood glucose levels of normal humans, DMEM-40 and DMEM-140 were defined as low and high glucose here.

### Phosphorylation of c-Jun and p53

**Figure 1:**
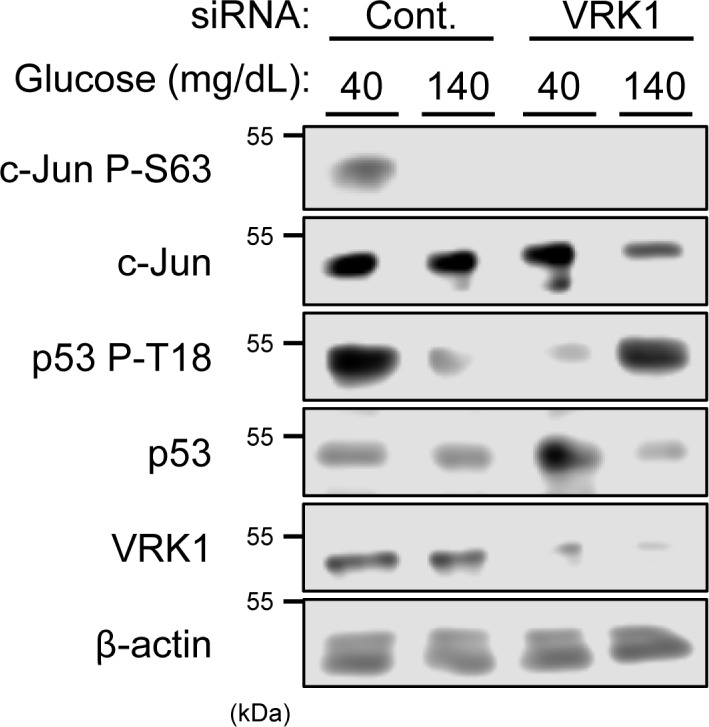
VRK1 activation by glucose stimulation Huh-7 cells were transfected with siVRK1 or control for 24 h in middle (100 mg/dl) glucose and, subsequently, in low (40 mg/dl) or high (140 mg/dl) glucose for 3 h. Whole extracts of these cells were prepared using urea buffer. c-Jun and p53 phosphorylation were analyzed by Western blotting using an anti-P-Ser63 c-Jun antibody and an anti-P-Thr18 p53 antibody, respectively. Protein expression levels of c-Jun, p53 or VRK1 were determined by Western blotting assay. Expression levels of β-actin were used for loading control.

Huh-7 cells were cultured in DMEM-40 (glucose: 40 mg/dl) or DMEM-140 (glucose: 140 mg/dl) medium. Huh-7 cells were treated with control siRNA or siRNA VRK1 to knock it down. Whole cell extracts were prepared for subsequent Western blot analysis to examine phosphorylation of c-Jun and p53 at Ser63 and Thr18, respectively. For both c-Jun and p53, phosphorylation observed with cells cultured in DMEM-40 was significantly attenuated by VRK1 knock down (Figure 1). This indicated that VRK1 phosphorylates these residues in low glucose. Unlike c-Jun, p53 displayed increased phosphorylation in VRK1 knockdown cells cultured in DMEM-140 medium. This implies that VRK1 prevents p53 from being phosphorylated in high glucose; the mechanism of this reaction remains unknown and will not be focused on here.

### Residues of VRK1 auto-phosphorylated

**Figure 2:**
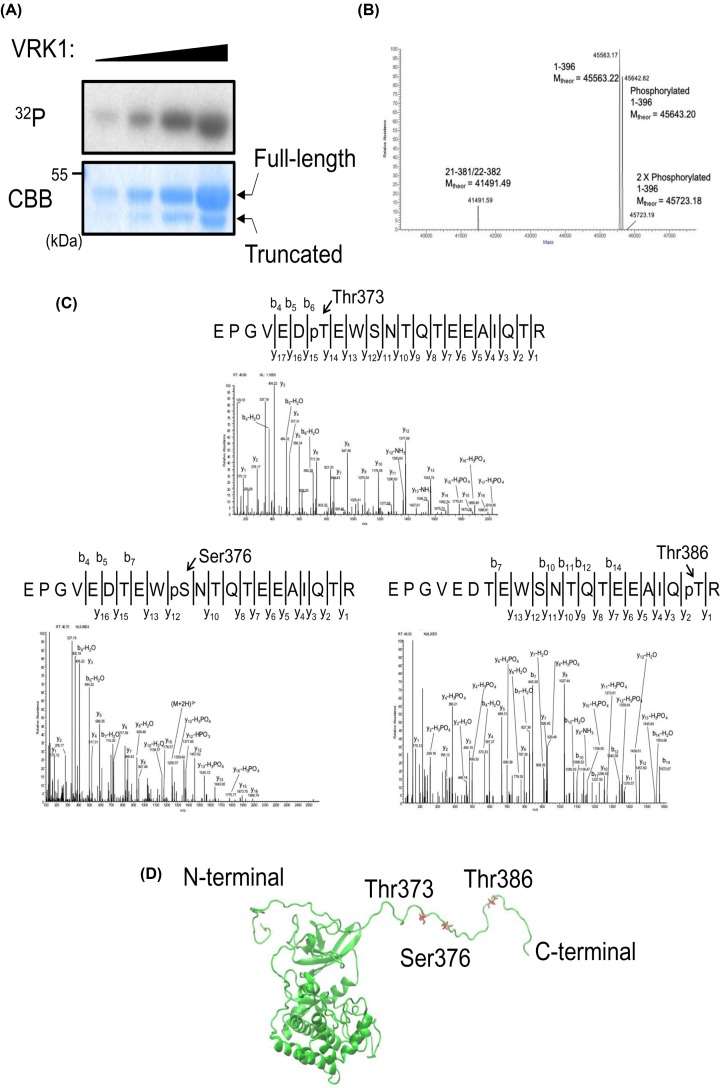
Determination of VRK1 phosphorylation residues (**A**) VRK1 auto-phosphorylation activity was analyzed by *in vitro* kinase assay. *Escherichia coli* expressed recombinant VRK1 was incubated with 32P-labeled ATP, electrophoresed on 10% SDS-polyacrylamide gel, and detected by autoradiography using x-ray film. (**B**) Purified, auto-phosphorylated VRK1 contains at least four species. Intact mass analysis with a high-resolution, high-mass accuracy orbitrap mass spectrometer showed full-length VRK1 as well as singly, and doubly phosphorylated forms though the doubly phosphorylated is at very low abundance. Additionally, a non-phosphorylated, truncated form of VRK1 was identified. This fragment matches in mass to either 21–381 or 22–382. (**C**) Phosphorylated residues of recombinant VRK1 were determined by mass spectrometry. Recombinant, auto-phosphorylated VRK1 was digested with trypsin and LC-MS was performed. Approximately 90% of the VRK1 sequence was observed and multiple sites of phosphorylation were observed. Tryptic peptide 59 (T59) was observed as phosphorylated and MSMS spectra show that this phosphorylation can localize to three unique sites at residues T373, S376, and T386. (**D**) The phosphorylation sites of VRK1 are labeled and colored red on the overall structure (PDB ID: 2rsv).

Recombinant VRK1 purified from *E. coli* cells was auto-phosphorylated in kinase buffer in the presence of ^32^P-ATP. VRK1 protein was separated on SDS-polyacrylamide gel and auto-radiographed (Figure 2A). On the other hand, a lower molecular weight band corresponding to a spontaneously-arising truncated form of VRK1 (presumably a proteolytic fragment generated by a protease(s) from *E. coli*) was not radiolabeled, indicating that truncation abolished auto-phosphorylation. Mass spectrometric analyses of auto-phosphorylated VRK1 showed excellent agreement with the expected theoretical mass of the recombinant construct (Figure 2B). Moreover, the intact 1–396 species was also observed as singly and doubly phosphorylated. Furthermore, the truncated species could easily be detected and corresponds to either residues 21–381 or 22–382. Notably, this truncated species was not observed to be phosphorylated by mass spectrometry. As both the N- and C-terminal regions of VRK1 are removed by proteolysis, it is ambiguous as to the contributions of each of these regions to the activity of VRK1. As others have shown that the C-terminus of VRK1 is important for its auto-phosphorylation activity [16] and since this truncation removed 14 or 15 residues from the C-terminus, auto-phosphorylation could be regulated by structural motif within this C-terminal region of the VRK1 molecule. Subsequently, mass spectrometric analysis identified Thr373, Ser376, and Thr386 as residues that are auto-phosphorylated (Figure 2C). These residues reside on the random coil C-terminal loop (Figure 2D). Ser376 and Thr386 are conserved in mouse VRK1 but not Thr373. Additionally, while analyzing VRK1 for phosphorylation, we observed more than 90% of VRK1 sequence including all the serines, threonines, and tyrosines in the N-terminus of the protein, but we did not observe any phosphorylation in this region.

### Phosphorylation of VRK1 in cells

**Figure 3:**
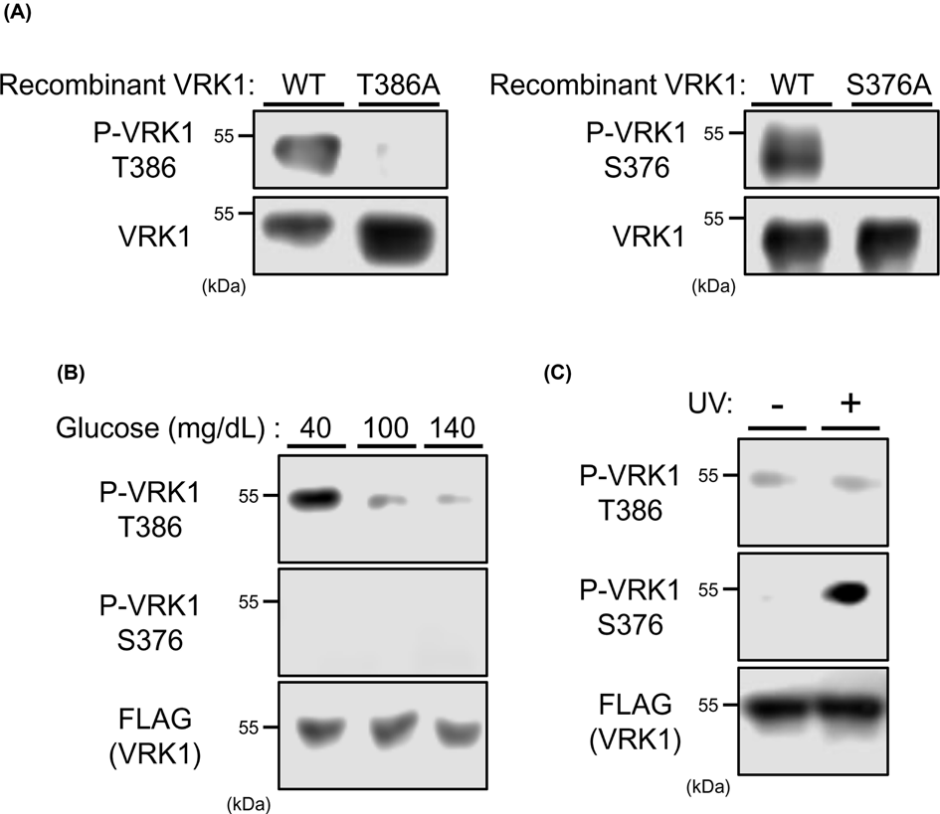
VRK1 phosphorylation in Huh-7 cells (**A**) Auto-phosphorylation of recombinant VRK1 was analyzed by Western blotting using anti-phospho specific antibodies for Ser376 or Thr386 of VRK1. (**B**) Huh-7 cells were expressed with FLAG-tagged VRK1 for 24 h in middle (100 mg/dl) glucose and, subsequently, in low (40 mg/dl), middle (100 mg/dl) or high (140 mg/dl) glucose for 3 h. Whole extracts of these cells were prepared using IP buffer for immunoprecipitation by anti-phospho VRK1 antibodies conjugated with Dynabeads Protein G. The resultant precipitates and whole cell extracts were analyzed by Western blotting for phospho-VRK1 and total FLAG-tagged VRK1, respectively, using an anti-FLAG HRP conjugated antibody. (**C**) After transfection with FLAG-tagged VRK1 for 24 h, Huh-7 cells were exposed to UV for 10 min. Whole extracts of these cells were prepared using IP buffer for immunoprecipitation by anti-phospho VRK1 antibodies conjugated with Dynabeads Protein G. The resultant precipitates and whole cell extracts were analyzed by Western blotting for phospho-VRK1 and total FLAG-tagged VRK1, respectively, using an anti-FLAG HRP conjugated antibody.

Phospho-peptide antibodies were produced to specifically detect phosphorylation of Thr386 and Ser376, respectively. First, to examine these antibodies, VRK1 WT and VRK1 T386A mutant were incubated in an *in vitro* kinase buffer and analyzed by Western blots. An anti-P-Thr386 peptide antibody detected VRK1 WT but not the mutant (Figure 3A). Similarly, an anti-P-Ser376 peptide antibody stained VRK1 WT but not VRK1 S376A mutant (Figure 3A). These assays confirmed that both residues were auto-phosphorylated. To examine these phosphorylation, FLAG-tagged VRK1 was ectopically expressed in Huh-7 cells in DMEM-40, DMEM-100, or DMEN-140 medium and analyzed by Western blots. Thr386 was phosphorylated in DMEM-40 medium but not in DMEM-100 and DMEM-140 media (Figure 3B). On the other hand, Ser376 was not phosphorylated in any of these media. Thus, Thr386 was phosphorylated in response to low glucose. The similar experiment was performed with Huh-7 cells exposed to UV, finding that Ser376, but not Thr386, was phosphorylated (Figure 3C). Hereafter, our present work will focus on Thr386.

### Regulation of The386 phosphorylation in cells

**Figure 4:**
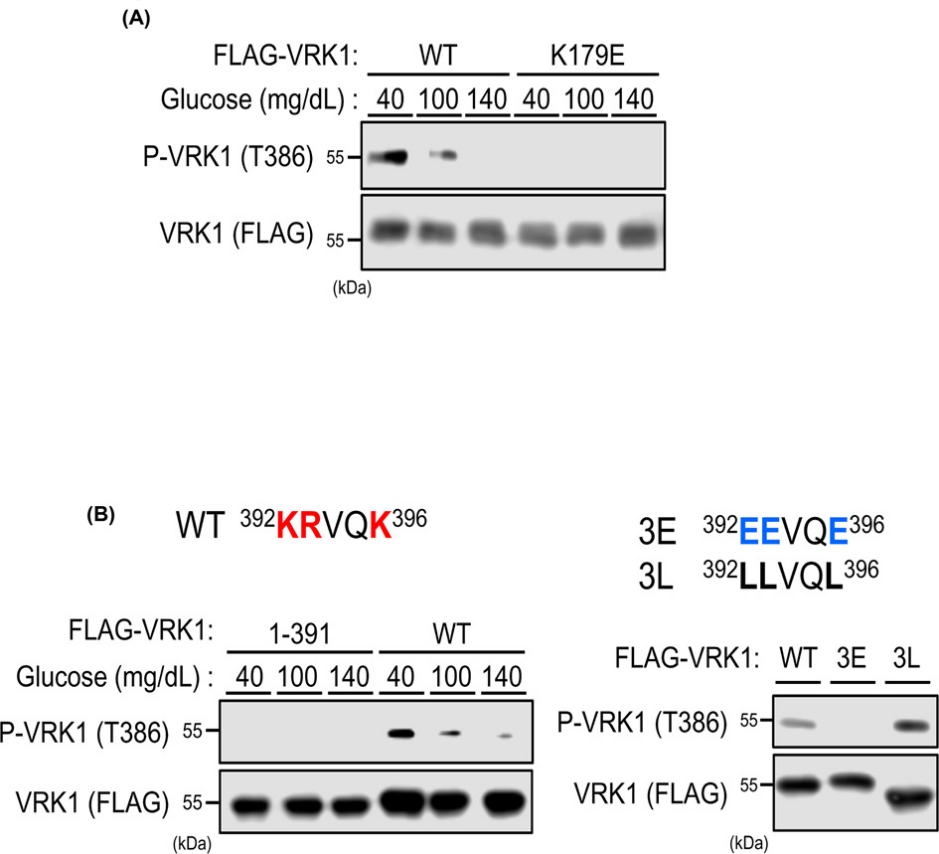
Regulation of VRK1 auto-phosphorylation (**A**) Huh-7 cells were expressed with FLAG-tagged VRK1-WT or kinase dead mutant (K179E) for 24 h in middle (100 mg/dl) glucose and, subsequently, in low (40 mg/dl), middle (100 mg/dl), or high (140 mg/dl) glucose for 3 h. Whole extracts of these cells were prepared using IP buffer for immunoprecipitation by anti-phospho VRK1 antibodies conjugated with Dynabeads Protein G. The resultant precipitates and whole cell extracts were analyzed by Western blotting for phospho-VRK1 and total FLAG-tagged VRK1, respectively, using an anti-FLAG HRP conjugated antibody. (**B**) Amino acid sequence of C-terminus of VRK1 (392-396)-WT or mutants. Positively and negatively charged amino acids are colored red and blue, respectively. Left: Huh-7 cells were expressed with FLAG-tagged VRK1-WT or C-terminus deletion mutant (1–391) for 24 h in middle (100 mg/dl) glucose and, subsequently, in low (40 mg/dl), middle (100 mg/dl), or high (140 mg/dl) glucose for 3 h. Whole extracts of these cells were prepared using IP buffer for immunoprecipitation by anti-phospho VRK1 antibodies conjugated with Dynabeads Protein G. The resultant precipitates and whole cell extracts were analyzed by Western blotting for phospho-VRK1 and total FLAG-tagged VRK1, respectively, using an anti-FLAG HRP conjugated antibody. Right: Huh-7 cells were expressed with FLAG-tagged VRK1-WT, negatively charged C-terminus mutant (3E) or reduction of C-terminus charging mutant (3L) for 24 h in middle (100 mg/dl) glucose. Whole extracts of these cells were prepared using IP buffer for immunoprecipitation by anti-phospho VRK1 antibodies conjugated with Dynabeads Protein G. The resultant precipitates and whole cell extracts were analyzed by Western blotting for phospho-VRK1 and total FLAG-tagged VRK1, respectively, using an anti-FLAG HRP conjugated antibody.

The active site mutation from Lys179 to glutamic acid has been shown to inactivate VRK1 auto-phosphorylation activity [8]. To examine whether Thr386 was auto-phosphorylated, VRK1 WT and VRK1 K179E mutant were ectopically expressed in Huh-7 cells cultured in DMEM-40 medium. Western blot analysis showed that Thr386 of this mutant was not phosphorylated (Figure 4A), thus indicating that VRK1 auto-catalyzed phosphorylation of Thr386 in cells. VRK1 has a short peptide at the C-terminus that is positively charged (Figure 4B). To examine if this peptide regulated VRK1 to phosphorylate Thr386, these five residues were removed from the C-terminus (VRK1 1-391). Ectopic VRK1 1–391 was found not to be phosphorylated at Thr386 in Huh-7 cells, while the corresponding residue of VRK1 WT was phosphorylated (Figure 4B, left). Furthermore, three positively charged residues were mutated to negatively charged glutamic acid (3E) or hydrophobic leucine (3L). These mutants were ectopically expressed in Huh-7 cells for subsequent Western blot analysis. The 3E was not phosphorylated at Thr386, while the 3L slightly increased this phosphorylation (Figure 4B, right). These observations indicate that these positively charged residues are important regulation at VRK1 auto-phosphorylation at Thr386 in a low glucose-dependent manner.

### Phosphorylation of c-Jun and p53 by VRK1 T386D in cells

**Figure 5:**
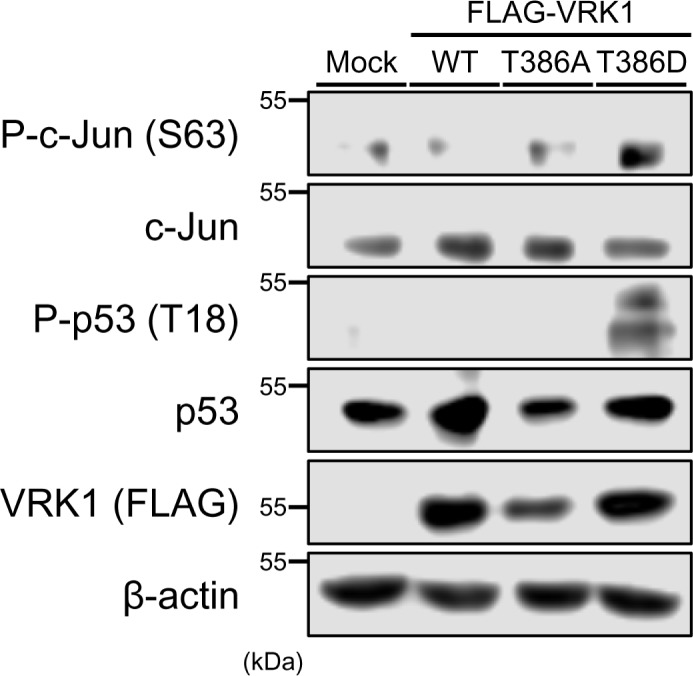
Regulation of VRK1 activity by phosphorylation Huh-7 cells were expressed with FLAG-tagged VRK1-WT, T386A, T386D, or mock for 24 h in high (140 mg/dl) glucose. Whole extracts of these cells were prepared using urea buffer. c-Jun and p53 phosphorylation were analyzed by Western blotting using an anti-P-Ser63 c-Jun antibody and an anti-P-Thr18 p53 antibody, respectively. Protein expression levels of c-Jun, p53 or FLAG-tagged VRK1 were determined by Western blotting. Expression levels of β-actin were used for loading control.

Phosphomimetic VRK1 T386D was utilized to examine whether VRK1 phosphorylated at Thr386, in fact, is the enzyme that phosphorylates c-Jun and/or p53. To this end, FLAG-tagged VRK1-WT, VRK1-T386A, or VRK1-T386D was ectopically expressed in Huh-7 cells cultured in DMEM-140 medium. Only when the VRK1 T386D was expressed, both c-Jun and p53 displayed increased levels of their phosphorylation (Figure 5). It appears that VRK1 is phosphorylated at Thr386 in response to low glucose, providing it with capability to phosphorylate c-Jun and p53.

### PKP2 as glucose signal to VRK1

**Figure 6:**
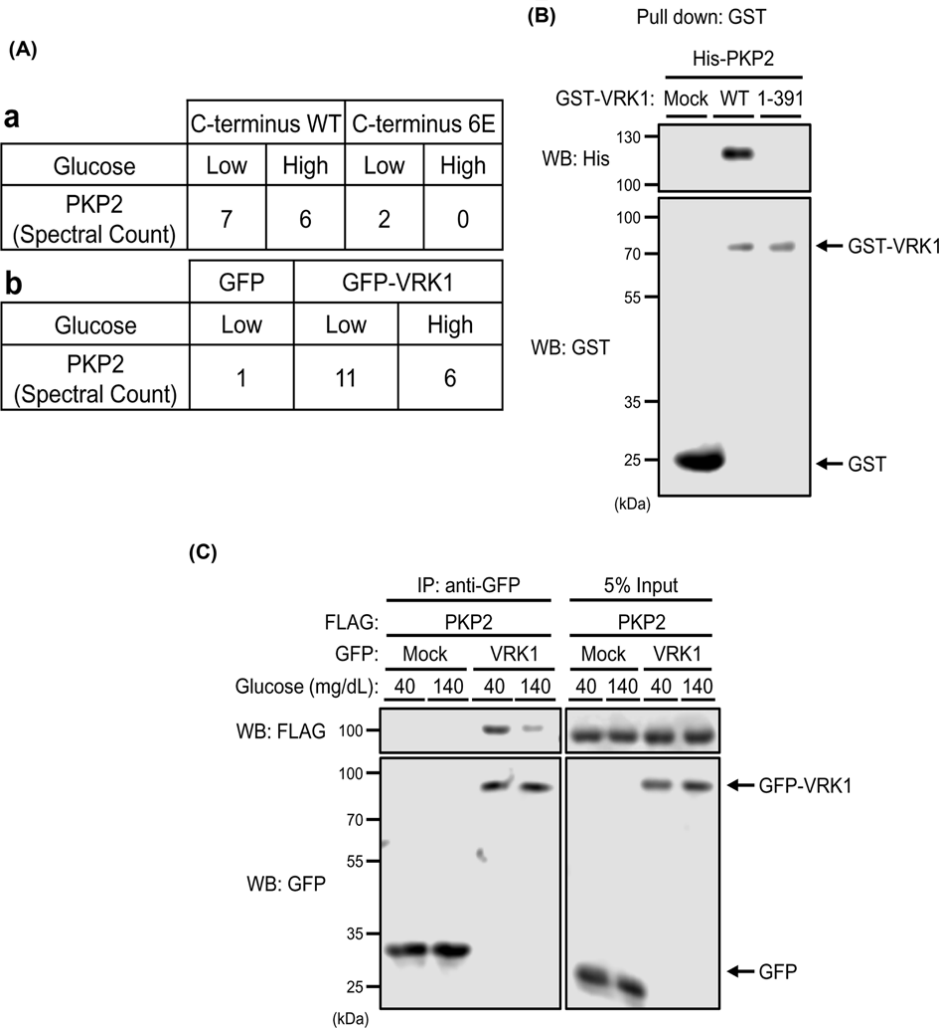
Protein–protein interaction between PKP2 and VRK1 (**A**) Table shows numbers of spectral counts detected for PKP2 in mass spectroscopy. a. Peptides specifically detected when the C-terminal peptide as a bait over its negatively charged mutant. b. Numbers of peptides recovered by GFP-tagged VRK1-WT as a bait over GFP. (**B**) Bacterially expressed His-tagged PKP2 was incubated with bacterially expressed GST-tagged VRK1-WT, 1–391 mutant or GST-conjugated glutathione resin. Protein bound resins were eluted and analyzed by Western blotting. (**C**) Huh-7 cells were co-expressed with FLAG-tagged PKP2 and GFP-tagged VRK1 or GFP for 24 h in middle (100 mg/dl) glucose and, subsequently, in low (40 mg/dl) or high (140 mg/dl) glucose for 3 h. Whole extracts from these cells were prepared by Co-IP buffer for immunoprecipitation by anti-GFP agarose beads. The resultant precipitates and whole cell extracts (as Input) were analyzed by Western blotting using an anti-FLAG HRP conjugated antibody or an anti-GFP HRP conjugated antibody.

VRK1 was activated to phosphorylate c-Jun and p53 in response to low glucose and this activation is regulated by the C-terminal peptide of the VRK1 molecule. To identify proteins that binds the C-terminal peptides, ^377^NTQTEEAIQTRSRTRKRVQK^396^ (WT) and negatively charged ^377^NTQTEEAIQTESETEEEVQE^396^ mutant (6E) were used as positive and negative baits, respectively, to pull down proteins, from whole cell extracts prepared from Huh-7 cells cultured in DMEM-40 or DMEM-140 medium. The resultant proteins were analyzed by mass spectrometry. Plakophilin-2 (PKP2) was identified with an aggregate summed MSMS score of 147.28 with 25.8% sequence coverage and 33 spectra representing 13 unique peptides. Higher numbers of PKP2 peptides recovered by the positive bait from the low glucose identified PKP2 as a protein that bind the C-terminal peptide specifically (Figure 6A,a). Similarly, GFP-tagged full-length VRK1 WT was used as bait to show PKP2 binding to VRK1 (Figure 6A,b). Pull-down assays were next employed to examine direct binding between PKP2 and VRK1. Recombinant GST-VRK1 and His-tagged PKP2 proteins were purified from *E. coli* cells and used for this assay. PKP2 pull-downed VRK1 but not VRK1 1–391. This supports direct binding of PKP2 to the C-terminal peptide of the VRK1 molecules. (Figure 6B). Subsequently, it was examined whether PKP2 and VRK1 formed a complex in Huh-7 cells. FLAG-tagged PKP2 and GFP-tagged VRK1 were co-expressed in Huh-7 cells cultured in DMEM-40 or DMEM-140 medium for subsequent co-immunoprecipitations. Co-precipitation was profoundly increased when cells were cultured in DMEM-40 medium (Figure 6C). These observations suggested that PKP2 binds VRK1 through the C-terminal peptide in cells in response to low glucose.

### PKP2 determines VRK1 substrate specificity

**Figure 7:**
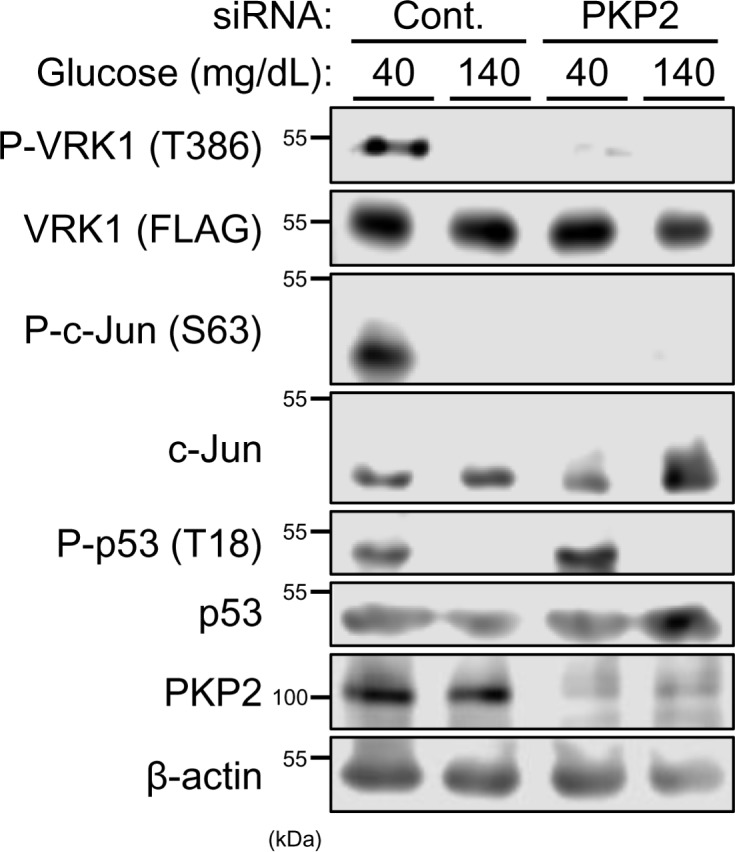
Regulation of VRK1 activity by PKP2 After transfection with siPKP2 or control for 24 h, Huh-7 cells were expressed with FLAG-tagged VRK1 for 24 h in middle (100 mg/dl) glucose and, subsequently, in low (40 mg/dl) or high (140 mg/dl) glucose for 3 h. Whole extracts of these cells were prepared using IP buffer. Phosphorylated FLAG-tagged VRK1 was immunoprecipitated by anti-phospho VRK1 antibodies conjugated with Dynabeads Protein G. The resultant precipitates and whole cell extracts were analyzed by Western blotting for phospho-VRK1 and total FLAG-tagged VRK1, respectively, using an anti-FLAG HRP conjugated antibody. c-Jun and p53 phosphorylation were analyzed by Western blotting using an anti-P-Ser63 c-Jun antibody and an anti-P-Thr18 p53 antibody, respectively. Protein expression levels of c-Jun, p53 or PKP2 were determined by Western blotting. Expression levels of β-actin were used for loading control.

PKP2 was knocked down by siRNA in Huh-7 cells cultured in either low or high glucose medium. Whole cell extracts were prepared and subjected to Western blot analysis (Figure 7 and Supplemental Figure SA). VRK1 was preferentially phosphorylated at Thr386 in cells cultured in low glucose medium. PKP2 knockdown decreased this phosphorylation. In correlating to the decreased auto-phosphorylation of VRK1, c-Jun also displayed decreased levels of phosphorylation at Ser63 (Figure 7). c-Jun phosphorylation was rescued by VRK1-T386D over-expression in siRNA PKP2-treated Huh7 cells (Supplemental Figure SA). On the other hand, PKP2 overexpression didn’t affect c-Jun phosphorylation induced by low glucose medium (Supplemental Figure SB). As opposed to c-Jun phosphorylation, PKP2 knockdown did not affect p53 phosphorylation (Figure 7). The overexpression of VRK1 T386D was decreased and increased p53 phosphorylation levels by low glucose medium and high glucose medium, respectively, in siRNA PKP2-transfected Huh7 cells (Supplemental Figure SA). These indicated that PKP2 activated VRK1 in Huh-7 cells cultured in a low glucose medium and determined its substrate specificity to phosphorylate c-Jun but not p53.

## Discussion

**Figure 8:**
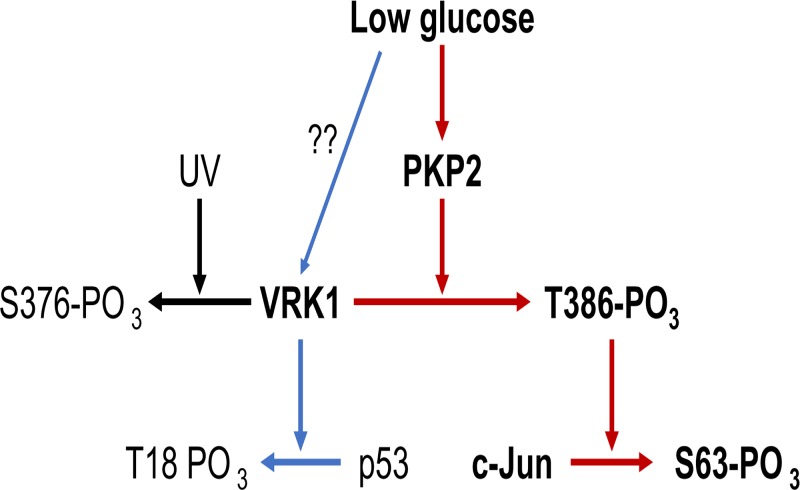
Schematic representation of VRK1 regulation and activation VRK1 auto-phosphorylated at Ser376 and Thr386 in response to UV-treatment induced DNA damage and low glucose signal, respectively, in Huh-7 cells. Thr386 phosphorylation by low glucose signal activates VRK1 to phosphorylate c-Jun and p53. PKP2 mediated this glucose signal to not only activate VRK1 but also determine its substrate specificity through binding to C-terminal peptide of VRK1. It is unclear the regulation mechanism of p53 phosphorylation by low glucose signal activated VRK1.

VRK1 phosphorylated at Thr386 is now characterized as the kinase that phosphorylates c-Jun and p53 in response to low glucose in Huh-7 cells. Cadherin PKP2 transduces low glucose signal to VRK1 by binding its C-terminal peptide motif, determining the substrate specificity of phosphorylated VRK1 toward c-Jun but not p53. An additional but unknown factor may be needed to regulate VRK1 phosphorylation of p53. For more than a decade since VRK1 was reported to phosphorylate c-Jun, its upstream signal has been a mystery. Now, glucose is defined to initiate this signal and PKP2 is characterized to transduce it to VRK1. Figure 8 depicts this glucose signaling through VRK1.

Previously, glucose was suggested to regulate VRK1, in which low glucose activates VRK1 to phosphorylate PXR at Ser350, inducing transcription of the PEPCK1 and gluconeogenesis in human hepatoma-derived HepG2 cells [12]. However, it remained unknown that residues of VRK1 are auto-phosphorylated when VRK1 is activated in to phosphorylate PXR response to low glucose. Now three residues are identified to be auto-phosphorylated in *in vitro* kinase assays: Thr373, Ser376, and Thr386, all of which reside near the C-terminus of the VRK1 molecule. Among them, Ser376 and Thr386 are conserved in both mouse and human VRK1s. Deletion of a C-terminal region that contains these phosphorylation residues was known to inactivate VRK1 auto-phosphorylation [16]. The reason for this inactivation is now attributed to the lack of phosphorylation. It may be that these residues can be targeted by various signals in addition to glucose, providing the experimental basis for future investigations. Mutations of these phosphorylation residues can be used to define VRK1 functions in *in vitro* as well as *in vivo* experimental models including animal models.

VRK1 becomes phosphorylated at different residues in response to different stimuli; Thr386 is phosphorylated in response to low glucose, while UV exposure induces Ser376 phosphorylation (Figure 8). These different responses suggest the presence of protein factors that differently regulate phosphorylation depending on different signals. In this, cadherin PKP2 is characterized as the low glucose transducer to VRK1 to be phosphorylated at Thr386 as well as to determine its substrate specificity to phosphorylate c-Jun. PKP2 is present in desmosomes and in the nucleus [17]. Since PKP2 directly binds VRK1 and both VRK1 and PKP2 are co-present in the nucleus, glucose can directly transduce its signal to PKP2 there. Alternatively, since glucose first contacts the cell membrane where PKP2 also resides, the signal could be initiated on PKP2 here to carry glucose signal to VRK1 in the nucleus. Nevertheless, the mechanism by which glucose transduces its signal remains an interesting question for future investigations. Unlike the case of c-Jun, p53 was phosphorylated by VRK1 in the absence of PKP2 in Huh-7 cells. Moreover, VRK1-T386D lowered the levels of p53 phosphorylation. These findings suggested that PKP2 activates VRK1 but does not determine its substrate specificity towards p53 and, possibly suggesting the presence of an additional protein and/or regulation of autophosphorylation status of VRK1 that determines p53 phosphorylation (Figure 8). Through potential regulatory proteins such as PKP2, VRK1 may acquire diverse capabilities to respond to various signals and regulate biological processes.

VRK1 regulates many biological processes, implicating it in disease pathologies such as UV-induced DNA damages. UV exposure specifically induces VRK1 phosphorylation at Ser376. Utilizing Ser376 as a target, the molecular mechanism by which VRK1 repairs DNA-damage can be investigated. VRK1-deficient mice of both males and females are infertile, which was considered to be caused by defects in spermatogenesis, oogenesis, or folliculogenesis [4,22,23]. In addition, VRK1 partial knockdown leads to a neurological impairment in mice [3]. Now, specific knockin mutations can be introduced to these phosphorylation residues to investigate the molecular mechanism of VRK1-regulated gametogenesis. Known mutation of the *VRK1* gene, p.W375X, which has been linked to development of neurodegenerative disease in human [24], truncates a C-terminal peptide of the VRK1 molecule that bears Ser376 and Thr386. This mutation enables us to examine that residues are phosphorylated in neurons during disease development. Mouse models can be generated by knocking in specific mutations to investigate the role of VRK1 in disease developments and its molecular mechanism.

VRK1 has been suggested to regulate hepatic metabolism through nuclear receptors PXR and FXR [11,12]. VRK1 phosphorylates PXR at Ser350 within the ligand-binding domain (LBD) in response to low glucose, increasing gluconeogenesis. VRK1 phosphorylates FXR at Ser154 within the DNA-binding domain (DBD) in the nucleus of hepatocytes of mouse livers. This phosphorylation is associated with ligand activation of FXR, the primary nuclear receptor that regulates bile acid metabolism. While residues that are phosphorylated to activate VRK1 remains unknown now, these can be determined by experiments of various VRK1 mutants replacing these Ser and Thr residues already identified. Ser350 of PXR and Ser154 of FXR are highly conserved as phosphorylation motifs within the LBD and DBD, respectively, of both human and mouse nuclear receptors [19]. VRK1 may phosphorylate many nuclear other receptors via the conserved motif far beyond PXR and FXR, regulating their functions. Nuclear receptors and stress factors such as c-Jun and p53 respond to common cell signals such as glucose and regulate the same metabolic pathways. It had been reported that nuclear receptors cross-talk with c-Jun or p53 [20–25]. Future investigation may find that VRK1 mediates these cross-talks by phosphorylating their conserved motifs in response to endo- and/or exogenous stimuli.

In conclusion, the auto-phosphorylated residues of VRK1 (Thr373, Ser376, Thr386) have been determined. VRK1 utilizes these residues to receive cell signals and transduces them to c-Jun and p53 in cells. Thr386 phosphorylation is defined as a low glucose signal that activates VRK1 to phosphorylate and activate c-Jun and p53. The low glucose-PKP2-VRK1-c-Jun pathway is established as the glucose stress signal pathway. These phosphorylation residues can be examined for their ability to influence the molecular mechanism by which VRK1 regulates normal physiology such as liver metabolism and gametogenesis and disease developments such as ALS.

## Supplementary Material

Supplemental Figure SA-SB

## Abbreviations

ALS: amyotrophic lateral sclerosis
CREB: cAMP response element binding protein
DBD: DNA-binding domain
FXR: farnesoid X receptor
IPTG: isopropyl-1-thio-D-galactopyranoside
LBD: ligand-binding domain
PKP2: plakophilin-2
PXR: pregnane x receptor
VRK1: vaccinia-related kinase 1
