# Supplementary material for: Phosphorylation of vaccinia-related kinase 1 at threonine 386 transduces glucose stress signal in human liver cells
Source: Biosci Rep. 2020 Apr 23;40(4):BSR20200498. doi: 10.1042/BSR20200498 (PMC7198045; doi:10.1042/BSR20200498)
Supplement: Supplemental Figure SA-SB [file BSR-2020-0498_supp.pdf]

Supplemental figure

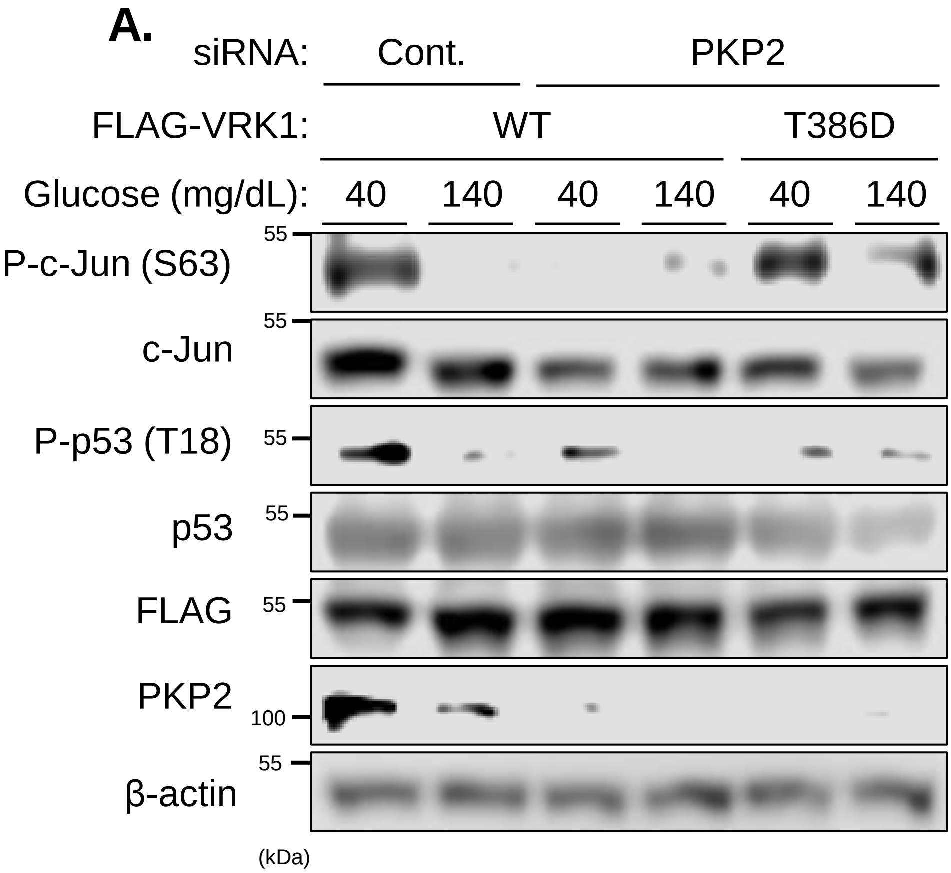

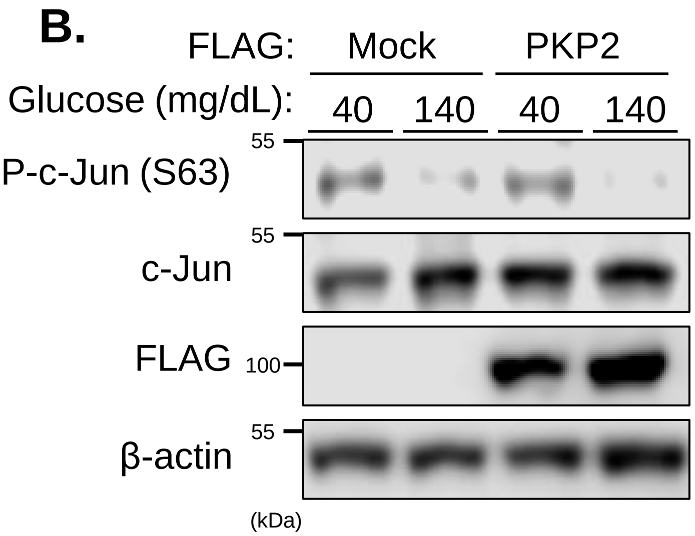

Supplemental Figure 1: (A) After transfection with siPKP2 or control for 24 hours, Huh-7 cells were expressed with FLAG-tagged VRK1-WT or T386D for 24 hours in middle (100 mg/dL) glucose and, subsequently, in low (40 mg/dL) or high (140 mg/dL) glucose for 3 hours. Whole extracts of these cells were prepared using urea buffer. c-Jun and p53 phosphorylation were analyzed by Western blotting using an anti-P-Ser63 c-Jun antibody and an anti-P-Thr18 p53 antibody, respectively. Protein expression levels of c-Jun, p53, FLAG or PKP2 were determined by Western blotting assay. Expression levels of β-actin were used for loading control. (B) Huh-7 cells were expressed with FLAG-tagged PKP2 or mock for 24 hours in middle (100 mg/dL) glucose and, subsequently, in low (40 mg/dL) or high (140 mg/dL) glucose for 3 hours.Whole extracts of these cells were prepared using urea buffer. c-Jun phosphorylation was analyzed by Western blotting using an anti-P-Ser63 c-Jun antibody. Protein expression levels of c-Jun or FLAG-tagged PKP2 were determined by Western blotting. Expression levels of β-actin were used for loading control.
